# Effectiveness of digital care platform CMyLife for patients with chronic myeloid leukemia: results of a patient-preference trial

**DOI:** 10.1186/s12913-023-09153-9

**Published:** 2023-03-08

**Authors:** Lynn Verweij, Geneviève I. C. G. Ector, Yolba Smit, Bas van Vlijmen, Bert A. van der Reijden, Rosella P. M. G. Hermens, Nicole M. A. Blijlevens

**Affiliations:** 1 Department of Hematology, Radboud Institute for Health Sciences, Radboud University Medical Center, Nijmegen, Netherlands; 2 Department of Pharmacy, Radboud University Medical Center, Nijmegen, Netherlands; 3 Department of Laboratory Medicine, Radboud Institute for Molecular Life Sciences, Radboud University Medical Center, Nijmegen, Netherlands; 4 Department of IQ Healthcare, Radboud Institute for Health Sciences, Radboud University Medical Center, Nijmegen, Netherlands

**Keywords:** Digital care platform, CML, Information provision, Patient empowerment, Medication compliance, Guideline adherence, Molecular monitoring, Quality of life

## Abstract

**Background:**

Two most important factors determining treatment success in chronic myeloid leukemia (CML) are adequate medication compliance and molecular monitoring albeit still being suboptimal. The CMyLife platform is an eHealth innovation, co-created with and for CML patients, aiming to improve their care, leading to an increased quality of life and the opportunity of hospital-free care.

**Objective:**

To explore the effectiveness of CMyLife in terms of information provision, patient empowerment, medication compliance, molecular monitoring, and quality of life.

**Methods:**

Effectiveness of CMyLife was explored using a patient-preference trial. Upon completion of the baseline questionnaire, participants actively used (intervention group) or did not actively use (questionnaire group) the CMyLife platform for at least 6 months, after which they completed the post-intervention questionnaire. Scores between the intervention group and the questionnaire group were compared with regard to the within-subject change between baseline and post-measurement using Generalized Estimating Equation models.

**Results:**

At baseline, 33 patients were enrolled in the questionnaire group and 75 in the intervention group. Online health information knowledge improved significantly when actively using CMyLife and patients felt more empowered. No significant improvements were found regarding medication compliance and molecular monitoring, which were already outstanding. Self-reported effectiveness showed that patients experienced that using CMyLife improved their medication compliance and helped them to oversee their molecular monitoring. Patients using CMyLife reported more symptoms but were better able to manage these.

**Conclusions:**

Since hospital-free care has shown to be feasible in time of the COVID-19 pandemic, eHealth-based innovations such as CMyLife could be a solution to maintain the quality of care and make current oncological health care services more sustainable.

**Trial registration:**

ClinicalTrials.gov NCT04595955, 22/10/2020.

**Supplementary Information:**

The online version contains supplementary material available at 10.1186/s12913-023-09153-9.

## Introduction

To meet the growing burden of chronic diseases, the World Health Organization reported that health care systems must reorganize and that innovative approaches are essential [1]. Cancer has become a notable proportion of these chronic diseases, as life expectancy of oncology patients has increased substantially due to new technologies, earlier diagnosis and better treatments [2, 3]. An example of such an oncologic chronic disease is chronic myeloid leukemia (CML). CML is characterized by translocation of chromosome 9 and 22, leading to the BCR-ABL1 mutation, which encodes for a constitutively active protein e.g. tyrosine kinase [4, 5]. Since tyrosine kinase inhibitors (TKIs) have been used as treatment the life expectancy of CML patients has increased. It approaches that of the general population, if patients have optimal responses to the treatment [6, 7].

The two most important factors determining treatment success are patients’ medication compliance and adequate molecular monitoring of BCR-ABL1 values according to evidence-based guidelines [8]. The BCR-ABL1 value is a disease-specific biomarker that accurately reflects the course of the disease and treatment response [8]. This monitoring of disease activity in accordance with guidelines is associated with a reduced risk of progression and mortality [9], improved TKI adherence [10], and lower health care costs [11]. Treatment-free remission has become an additional treatment goal, where TKI treatment can be safely discontinued without relapsing [12]. However, guideline adherence is still suboptimal and tends to be lower in hospitals, which are less experienced in treating CML patients [9, 13]. Hence, patients could play an important role in guarding their own molecular values.

This shift to more involved and empowered patients, and ultimately to hospital-free care, needs a patient-centered approach, based on eHealth [14–18]. eHealth could provide patients with tools to oversee and guard the progress and accuracy of their treatment. If they have any questions they could get in touch with their health care provider (HCP) to monitor their treatment. Therefore, eHealth could be a promising medium for patients to easily monitor their molecular levels from home. Besides, eHealth enables patients to monitor their medication compliance, provides patients with access to care efficiently, enables interactive patient-provider communication, and helps them to access and understand medical information more readily [17, 18]. Hence, eHealth seems to have considerable potential and added value, as it may give patients the chance to get sufficient information and care from home and only visit the hospital when necessary. Feasibility of eHealth platforms is extensively investigated, but insight into their effectiveness in haematological malignancies is scarce.

CMyLife is an example of such an eHealth innovation and was co-created with and for CML patients [19]. CMyLife aims to provide CML patients with tools and knowledge to control their care process and improve their medication compliance and molecular monitoring. This could eventually lead to increased quality of life and opportunity of hospital-free care. The aim of this study is to explore the effectiveness of the CMyLife platform in terms of information provision, patient empowerment, medication compliance, molecular monitoring, and quality of life of CML patients.

## Methods

### Design

A patient-preference trial was performed in order to gain insights into the effectiveness of CMyLife in terms of information provision, patient empowerment, medication compliance, molecular monitoring of BCR-ABL1 values, and quality of life of CML patients. Patients could choose between actively or not actively using CMyLife, including a pre- and post-measurement. The study period lasted from July 2019 to October 2020. Given the nature of this study and the low impact on participants, the Medical Research Involving Human Subjects Act (Dutch: WMO) does not apply, as confirmed by the institutional Medical Ethical Committee ‘CMO Regio Arnhem-Nijmegen’. The trail was registered at ClinicalTrials.gov at 22/10/2020 (registration number: NCT04595955).

### Study population

HCPs approached their patients (who visited them for an appointment within the study period) for participation in this study in five academic and seven large non-academic hospitals, spread over the Netherlands. In addition, participants were recruited through the website of the Dutch patient advocate association, called Hematon, and via the CMyLife website [20]. Upon participation in the study, all participants signed informed consent. The study population consisted of patients in chronic phase CML who were treated with first or second line TKIs. Patients treated with second line TKIs were only allowed to participate if they had switched TKIs as a result of intolerance, not because of treatment failure. Patients were excluded if they were in treatment-free remission, in acceleration phase, in blast crisis, or if pregnancy was planned in the study period.

Patient preference was leading in determining group enrolment. Patients who agreed to actively use CMyLife for at least 6 months were enrolled in the treatment group; patients who did not were enrolled in the questionnaire group. Active use in the intervention group comprised receival of adequate support from the CMyLife-team. The support consisted of an instruction package and a kick-off workshop to inform patients about how, when, and why they could and should use CMyLife. The CMyLife-team was reachable for questions during the day and offered all users to help them install the platform. Also, the CMyLife-team gathered feedback from users about if their support was adequate. Patients in the questionnaire group were aware of the existence of CMyLife and the website remained accessible for them. However, they received no support. After enrolment participants received a written baseline questionnaire by mail. Upon completion of the baseline questionnaire, participants actively used (intervention group) or did not actively use (questionnaire group) CMyLife for at least 6 months, after which they completed the post-intervention questionnaire.

### Intervention

Development and features of the CMyLife platform were described in detail elsewhere [19]. CMyLife facilitates CML patients with a website, medication app, guideline app, and a personal health environment. All data were secured and conform the General Data Protection Regulation. In accordance with the Dutch security guideline (NEN7510), features containing data from the hospital’s electronic health record (for example, laboratory results) were secured with a two-step validation with a token received via an SMS text message.

The website provides accurate and easy to understand information about the disease, medication, guidelines, side effects, and the effect on daily life (work, sports, mortgage etc.). It is provided with a question and answer functionality, where patients can receive answers from specialized haematologists and pharmacists. In addition, patients (in the intervention group) can communicate with other patients (in the intervention group), to share information or ask questions (via a password protected forum).

The medication app [21] is used to optimize medication compliance and supports patients in preparing the consultation with their HCP by allowing patients to set medication alarms, register their medication intake, request for repeat medication prescriptions and read the information leaflet of the medication they are taking. In addition, they can log side effects they experience, which can be shared with their HCPs through their personal health environment. The medication app also includes an option that enables patients to record the consultation with their HCPs.

The guideline app [22] displays scheduled blood checks and appointments with haematologists, sends monitoring reminders, and shows an understandable explanation of patients’ BCR-ABL1 values and of the Dutch treatment guidelines. This enables patients to compare their values to the guideline’s targets, and when they differ, act accordingly.

Patients can save their own medical records in their personal health environment, consisting of a Patient Knows Best portal [23]. They can determine which information they store and with whom they share it (adjustable by turning off or on their informed consent). For example, they can share side effects with their HCPs in order to discuss them.

### Data collection

At baseline, all participants completed a survey containing patient characteristics (i.e. sociodemographics, CML related information) and Dutch translated and validated questionnaires (Additional file 1) exploring the effectiveness of CMyLife in terms of information provision, patient empowerment, medication compliance, molecular monitoring and quality of life. Six months later, patients completed the post-intervention questionnaire containing the same validated questionnaires. The post-intervention questionnaire of patients in the intervention group included additional questions about self-reported effectiveness. Regarding information provision, familiarity with CML related concepts (Philadelphia chromosome, BCR-ABL1, tyrosine kinase inhibitor, remission, log reduction (for BCR-ABL1), hematological response/remission, cytogenetic response/remission, molecular response/remission, major molecular remission, complete cytogenetic remission, treatment-free remission, Hematon) was rated on a scale from 1 to 6. Information provision, eHealth literacy (patients’ ability to find and evaluate online health information), patient empowerment and medication compliance were measured using the validated questionnaires EORTC QLQ-INFO25 [24], eHealth Literacy Scale (eHEALS) [25], Patient Activation Measure (PAM) [26–28], and the Medication Adherence Rating Scale (MARS) [29, 30], respectively. The MARS was supplemented with patients’ self-reported effectiveness on medication compliance in the post-measurement of patients in the intervention group. Adequate molecular monitoring was measured using the frequency of hospital visits in the past 12 months and was supplemented with patients’ self-reported effectiveness on their molecular monitoring in the post-measurement of patients in the intervention group. Additional file 2 describes questions and interpretation of the self-reported effectiveness on medication compliance and molecular monitoring. Quality of life and disease-specific quality of life were measured using the EORTC QLQ-C30 [31], and the EORTC QLQ-CML24 [32], respectively. Table 1 shows an overview of outcomes with corresponding measures used in this study.

**Table 1:** Overview of outcomes and corresponding measures used

Outcome	Measure
Information provision	Familiarity with CML related concepts EORTC QLQ-INFO25^a^ [24]
eHealth literacy	eHealth Literacy Scale [25]
Patient empowerment	Patient Activation Measure [26–28]
Medication compliance	Medication Adherence Rating Scale [29, 30] Self-reported effectiveness on medication compliance ^b^
Molecular monitoring	Frequency of hospital visits in the past 12 months Self-reported effectiveness on molecular monitoring ^c^
Quality of life	EORTC QLQ-C30^d^ [31] EORTC QLQ-CML24^e^ [32]

^a^European Organization for Research and Treatment of Cancer Quality of Life Questionnaire Information 25-item, ^b^Questions and interpretation of self-reported effectiveness on medication compliance is described in Additional file 2, ^c^Questions and interpretation of self-reported effectiveness on molecular monitoring is described in Additional file 2. ^d^European Organization for Research and Treatment of Cancer Quality of Life Questionnaire Core 30-item, ^e^European Organization for Research and Treatment of Cancer Quality of Life Questionnaire Chronic Myeloid Leukemia 24-item

### Analysis

Measurements were processed anonymously, and analyses were performed with SPSS version 25 (IBM Corp). Descriptive statistics were used to describe basic features of participants. Attrition bias was evaluated by comparing patient characteristics between baseline and post-measurements. The EORTC QLQ-INFO25, eHEALS, PAM, MARS, EORTC QLQ-C30, and EORTC QLQ-CML24 were scored according to their manuals. In addition, all individual items on EORTC QLQ-INFO25 and EORTC QLQ-CML24 were analysed to get better insight in information gaps in CMyLife and in symptoms and problems of CML patients to improve CMyLife. Effectiveness of CMyLife was evaluated by comparing scores between the intervention and questionnaire group with regard to within-subject change between baseline and post-measurement using Generalized Estimating Equation models. If basic characteristics differed significantly between groups, *P*-values were corrected. Except for self-reported effectiveness, all differences shown in the results are referring to differences between intervention and questionnaire groups with regard to within-subject change between baseline and post-measurement.

## Results

### Patient characteristics

**Fig. 1:**
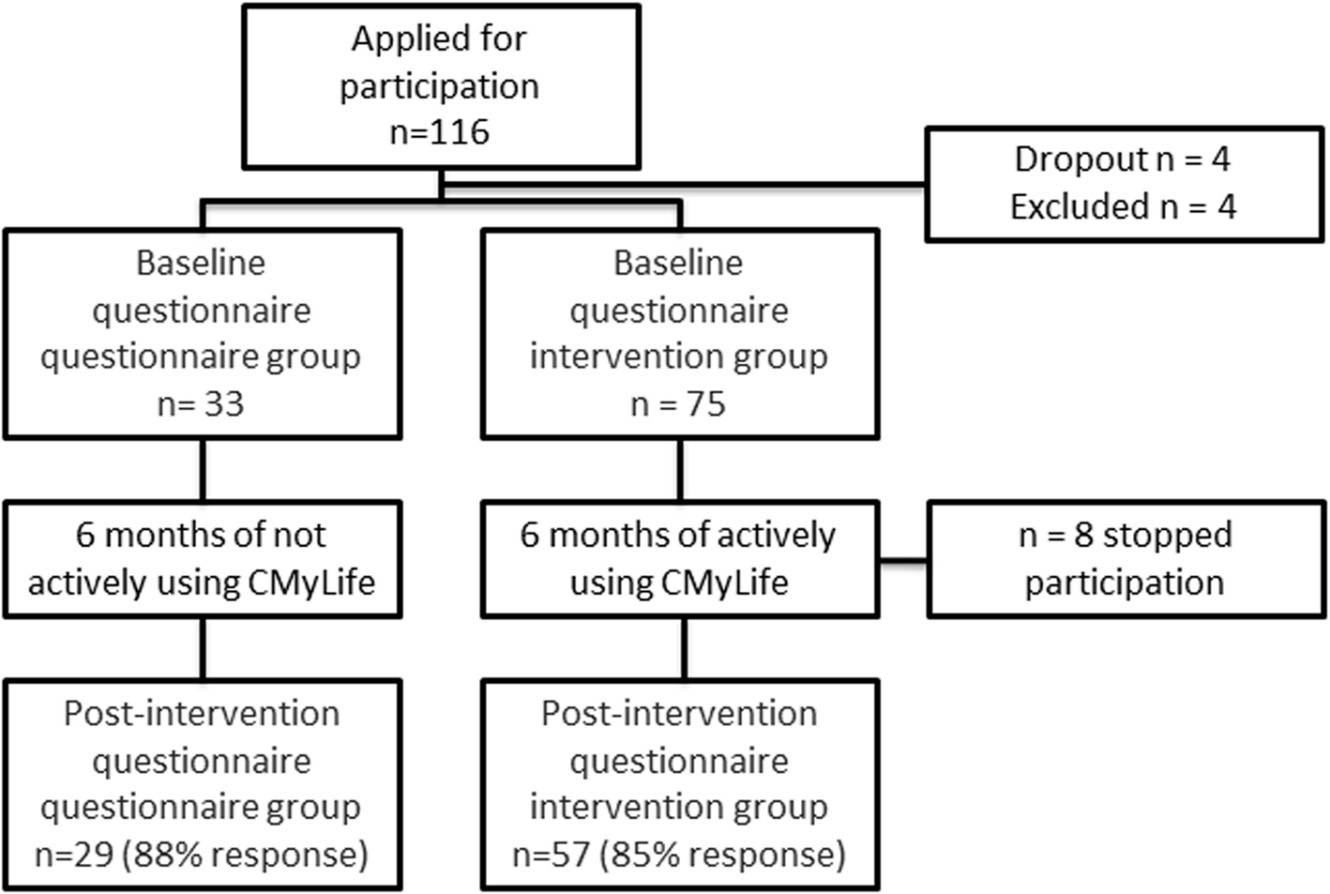
Flowchart study population

In total, 116 CML patients applied to participate in this study. Four patients dropped out, and four patients were excluded (stopped or never used TKIs). At baseline, 33 patients were enrolled in the questionnaire group and 75 in the intervention group. After 6 months, 29 patients filled in the post-intervention questionnaire and four patients did not return their answers (88% response) in the questionnaire group. During the six-month study period, eight patients in the intervention group stopped their participation. After 6 months, 57 patients filled in the post-intervention questionnaire and ten patients did not return their answers (85% response). Figure 1 shows the flowchart of the study. Attrition bias between pre- and post-measurements was evaluated and not detected. Table 2 summarises patient characteristics. In the questionnaire group, 64% were female, and 64% were aged ≥65. In the intervention group, 38% were female, and 40% were aged ≥65. The percentage of patients that used CMyLife before participating in this study was comparable between groups. Gender, age, education, and whether patients had previously been treated with TKIs differed significantly between groups (Table 2). Therefore, the *P*-values were corrected for these characteristics.

**Table 2:** Baseline characteristics for CML patients

Baseline characteristics	Questionnaire group (*n* = 33)	Intervention group (*n* = 75)	*P*-value
**Gender, n (%)**			**.013**
Male	12 (36.4)	46 (62.2)	
Female	21 (63.6)	28 (37.8)	
**Age (years), n (%)**			**.022**
18–64	12 (36.4)	44 (60.3)	
65 and older	21 (63.6)	29 (39.7)	
**Education level, n (%)**			**.000**
High^a^	10 (30.3)	39 (52.7)	
Middle^b^	7 (21.2)	26 (35.1)	
Low^c^	16 (48.5)	9 (12.2)	
Years since diagnosis, (mean ± SD)	6.7 ± 5.2	6.3 ± 6.2	.724
Previously used CMyLife, n (%)			.915
Yes, website	12 (36.4)	25 (33.3)	
Yes, medication app/ guideline app/otherwise	6 (18.2)	16 (21.3)	
No, not yet	14 (42.4)	31 (41.3)	
Currently used TKI, n (%)			.327
Yes, imatinib	16 (45.7)	44 (60.3)	
Yes, Otherwise^d^	16 (45.7)	29 (39.7)	
**Previously treated with TKI, n (%)**			**.007**
Yes	20 (62.5)	25 (34.2)	
No	11 (37.5)	48 (65.8)	
Hospital visits in the past 12 months, n (%)			.186
Zero	0 (0.0)	2 (2.7)	
1 time	3 (9.1)	2 (2.7)	
2 times	0 (0.0)	6 (8.1)	
3 times or more	30 (90.8)	64 (86.5)	

^a^high education level: higher vocational education, academic education. ^b^middle education level: secondary vocational education, higher general secondary education, pre-university education. ^c^low education level: primary education, lower general secondary education, preparatory secondary vocational education. ^d^Mostly dasatinib and nilotinib

### Information provision

During the study period, patients in the intervention group became significantly more familiar with patient association Hematon compared to patients in the questionnaire group (*P* = .023). For the other concepts, no statistically significant differences were found between the groups. However, in the intervention group, a clear trend towards better familiarity with disease related concepts was seen (Additional file 3).

Table 3 shows the percentage of patients who reported to receive no or little information on all individual information items. After the study period, information on “additional help outside the hospital” scored significantly better in the intervention group (*P* = .025). Several items showed a trend towards better information provision in the intervention group when compared to the questionnaire group (e.g. diagnosis, extent spread, possible causes of the disease, and expected effects and possible side effects of treatment). Table 3 also shows scores on perceived information provision on different EORTC-scales. There was no significant difference between the two groups although all information scales show a trend towards better information provision in the intervention group.

**Table 3:** Percentage of CML patients who perceived to receive no or little information per item of the EORTC QLQ-INFO25 and mean scores (SD) on information provision scales of the EORTC QLQ-INFO25, questionnaire vs intervention group

	**Questionnaire group**			**Intervention group**			
**Percentage of cml patients who perceived to receive no or little information about:**	**Pre (*n*** **= 33) %**	**Post (*n*** **= 29) %**	**Δ**	**Pre (*n*** **= 75) %**	**Post (*n*** **= 57) %**	**Δ**	**Corrected** ***P*-value**
The diagnosis of your disease	30.3	31.0	0.7	26.4	17.9	−8.5	.248
The extent (spread) of your disease	42.4	55.2	12.8	41.9	33.9	−8	.133
The possible causes of your disease	68.8	65.5	−3.3	64.9	55.4	−9.5	.505
Whether the disease is under control	18.2	17.2	−1	17.6	12.5	−5.1	.281
The purpose of any medical tests you have had or may undergo	27.3	31.0	3.7	20.3	16.1	−4.2	.448
The procedures of the medical tests	24.2	24.1	−0.1	23.0	16.4	−6.6	.608
The results of the medical tests you have already received	18.2	10.3	−7.9	12.2	9.1	−3.1	.622
The medical treatment	18.2	13.8	−4.4	13.5	9.1	− 4.4	.391
The expected benefit of the treatment	30.2	27.6	−2.6	18.9	9.1	−9.8	.106
The possible side-effects of your treatment	54.5	35.7	−18.8	51.4	27.3	−24.1	.688
The expected effects of the treatment on disease symptoms	48.5	34.5	−14	41.9	27.3	−14.6	.877
The effects of the treatment on social and family life	87.9	72.4	−15.5	81.1	72.7	−8.4	.529
The effect of the treatment on sexual activity	83.9	82.8	−1.1	90.5	88.9	−1.6	.940
**Additional help outside the hospita**l	**90.9**	**93.1**	**2.2**	**97.2**	**85.5**	**−11.7**	**.025**
Rehabilitation services	90.9	92.9	2	94.5	87.3	−7.2	.155
Aspects of managing your illness at home	93.9	86.2	−7.7	87.7	85.5	−2.2	.357
Possible professional psychological support	93.8	96.6	2.8	84.9	78.6	−6.3	.163
Different locations of care	84.4	93.1	8.7	89.0	85.5	−3.5	.155
Things that you can do to help yourself get well	75.0	78.6	3.6	78.1	78.2	0.1	.402
**EORTC-QLQ-INFO25 scales**^**a**^	**Questionnaire group**			**Intervention group**			
	**Pre (*n*** **= 33)** **Mean (SD)**	**Post (*n*** **= 29)** **Mean (SD)**	**Δ**	**Pre (*n*** **= 75)** **Mean (SD)**	**Post (*n*** **= 57)** **Mean (SD)**	**Δ**	**Corrected** ***P*-value**
Information about							
Disease	58.8 (24.1)	57.2 (22.5)	−1.6	57.1 (21.1)	63.2 (20.6)	6.1	.115
Medical tests	71.7 (24.2)	67.8 (20.6)	−3.9	69.7 (22.5)	70.0 (22.2)	0.3	.442
Treatments	48.1 (18.4)	53.8 (21.7)	5.7	50.2 (19.1)	55.4 (18.1)	5.2	.854
Other services	12.4 (18.1)	13.2 (19.7)	0.8	16.4 (18.6)	22.3 (25.6)	5.9	.252
Different location of care facilities	18.7 (25.3)	10.3 (20.1)	−8.4	16.9 (24.3)	20.0 (30.5)	3.1	.052
How to help yourself	29.2 (31.4)	29.8 (30.5)	0.6	30.6 (26.5)	33.3 (30.1)	2.7	.513
Received written information	68.8 (47.1)	67.9 (47.6)	−0.9	70.3 (46.0)	71.4 (45.6)	1.1	.884
Received cd/video	6.06 (24.2)	10.3 (31.0)	4.24	5.48 (22.9)	7.14 (26.0)	1.66	.538
Satisfaction with amount of information	43.4 (25.7)	41.4 (21.2)	−2	38.4 (23.4)	36.4 (20.6)	−2	.865
Wish to receive more info	33.3 (47.9)	31.0 (47.1)	−2.3	45.9 (50.2)	22.8 (42.3)	−23.1	.106
Wish to receive less info	66.7 (47.9)	69.0 (47.1)	2.3	54.1 (50.2)	77.8 (42.0)	23.7	.105
Helpfulness of information	31.3 (26.3)	34.5 (26.4)	3.2	31.1 (21.8)	32.1 (22.2)	1	.222
Global score	46.3 (16.1)	46.7 (17.0)	0.4	47.1 (15.9)	51.2 (16.5)	4.1	.161

*P*-value shows the difference between intervention and questionnaire with regard to the within-subject change between baseline and post-measurement

Δ = difference between pre- and post-measurements

^a^Scores between 0 and 100, higher scores indicate more/better information and satisfaction

### eHealth literacy

Table 4 shows mean scores on eHealth literacy. Results from the eHEALS showed that both groups had comparable competences before the study period. After the study period, patients’ knowledge on where to find helpful health resources on the internet was significantly improved in the intervention group (*P* = .032). Furthermore, the intervention group showed a trend towards better eHealth literacy after the study period.

**Table 4:** Mean scores (SD) on eHealth Literacy (eHEALS)^a^ per item questionnaire vs intervention group

	Questionnaire group			Intervention group			
Item	Pre (***n*** = 33) Mean (SD)	Post (***n*** = 29) Mean (SD)	Δ	Pre (***n*** = 75) Mean (SD)	Post (***n*** = 57) Mean (SD)	Δ	Corrected ***P***-value
I know what health resources are available on the Internet	4.03 (0.67)	3.86 (0.79)	−0.17	3.73 (0.97)	3.95 (0.77)	0.22	.076
**I know where to find helpful health resources on the Internet**	**4.03 (0.73)**	**3.79 (0.83)**	**−0.24**	**3.77 (1.04)**	**4.04 (0.69)**	**0.27**	**.032**
I know how to find helpful health resources on the Internet	3.88 (0.82)	3.83 (0.85)	−0.05	3.85 (0.92)	4.04 (0.69)	0.19	.424
I know how to use the Internet to answer my health questions	3.67 (0.82)	3.79 (0.68)	0.12	3.69 (0.98)	3.95 (0.70)	0.26	.543
I know how to use the health information I find on the Internet to help me	3.48 (0.94)	3.59 (0.73)	0.11	3.46 (1.00)	3.77 (0.76)	0.31	.464
I have the skills I need to evaluate the health resources I find on the Internet	3.69 (1.03)	3.86 (0.79)	0.17	3.58 (0.97)	3.96 (0.76)	0.38	.437
I can tell high-quality from low-quality health resources on the Internet	3.47 (1.05)	3.69 (0.89)	0.22	3.46 (1.05)	3.84 (0.89)	0.38	.703
I feel confident in using information from the Internet to make health decisions	3.31 (1.15)	3.36 (0.91)	0.05	3.26 (1.10)	3.71 (0.89)	0.45	.110

*P*-value shows the difference between intervention and questionnaire with regard to the within-subject change between baseline and post-measurement

Δ = difference between pre- and post-measurements

^a^Scores between 0 and 5 with higher scores indicating better eHealth literacy

### Patient empowerment

**Fig. 2:**
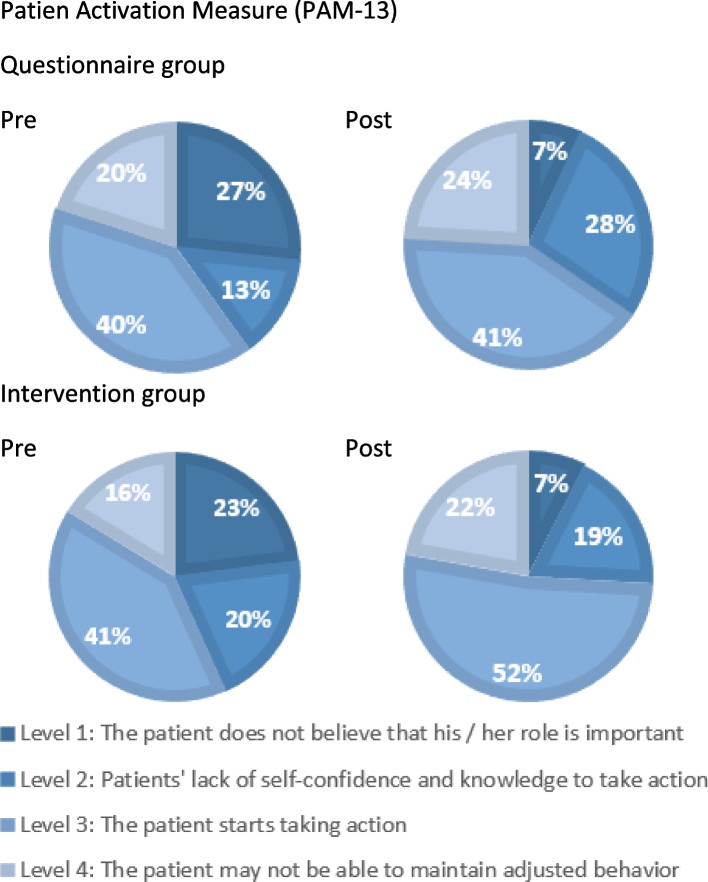
Patient empowerment levels (PAM-13 NL) questionnaire vs intervention group

Figure 2 shows scores on patient empowerment. At baseline, 40% of patients in the questionnaire group did not believe in their own role in treating their CML or did not have trust or knowledge to take action (level 1–2). After the study period, 35% of them was level 1 or 2. For the intervention group, these rates were 43 and 26%, respectively.

Moreover, there was no significant difference in the standardized activation score between the intervention and the questionnaire group (*P* = .139). In the intervention group, a trend towards increased patient empowerment was observed. The mean PAM score of the questionnaire group increased from 60.5 (SD = 14.6) to 62.6 (SD = 13.2) whereas the intervention group increased from 58.5 (SD = 13.7) to 64.0 (SD = 12.4).

### Medication compliance

No statistical difference was found in mean total score of patients’ medication compliance between groups (*P* = .922). Mean total score of the questionnaire group increased from 5.3 (SD = 0.92) to 5.4 (SD = 1.00) whereas the intervention group increased from 5.4 (SD = 0.54) to 5.5 (SD = 0.82) after using CMyLife. Patients’ self-reported effectiveness showed that 34.2% of patients in the intervention group reported that due to using the medication app they knew better why taking their medication properly is important. Additionally, 47.4% felt more motivated to take medication properly and 28.9% had started to think differently about how important it is. Using the medication app stimulated 21.0% of patients to search for help if necessary, it improved medication compliance of 42.1% of patients, and 52.7% of them felt less insecure about it.

### Molecular monitoring according to guidelines

In the questionnaire group, 90.8% of patients visited the hospital three times or more at baseline versus 86.2% in the post-measurement. In the intervention group, these rates decreased from 86.2 to 78.9%. No statistical differences were found between the groups (*P* = .685). In the self-reported effectiveness questionnaire, 44.0% of patients in the intervention group indicated that they were more aware of when they needed to get their BCR-ABL1 values checked, and 20.0% felt less insecure about it after using the guideline app. In addition, 20.0% of patients felt more motivated to get their BCR-ABL1 values checked timely when using the guideline app, and it stimulated 32.0% to contact their HCP if their response to treatment was not sufficient according to guidelines. Forty percent of patients knew when treatment response was good and 20.0% of patients started to think differently about how important it is to get their BCR-ABL1 values checked in time. Lastly, 32.0% of patients reported to have more insight into their BCR-ABL1 values and its course.

### Quality of life

Table 5 shows scores of the EORTC-QLQ-C30 and EORTC-QLQ-CML24 questionnaires. On the scales of the EORTC-QLQ-C30 no significant differences between the pre- and post-measurement of the groups were reported. Regarding the EORTC-QLQ-CML24, during the study period, individual items showed that patients in the intervention group had a significant increase in experiencing treatment as burdensome, compared to patients in the questionnaire group (*P* = .043) (Table 5). Also, on the scales of the EORTC-QLQ-CML24, a significant decrease was reported on impact on daily life (*P* = .043) and an increase in symptom burden (*P* = .013) in the intervention group compared to the questionnaire group (Table 5).

**Table 5:** Mean scores (SD) from the EORTC-QLQ-C30 and the EORTC-QLQ-CML24 scales and percentage of patients experiencing moderate to severe burden of disease specific complaints (EORTC QLQ-CML24): questionnaire vs intervention group

	**Questionnaire group**			**Intervention group**			
**Scales**	**Pre (*n*** **= 33)** **Mean (SD)**	**Post (*n*** **= 29)** **Mean (SD)**	**Δ**	**Pre (*n*** **= 75)** **Mean (SD)**	**Post (*n*** **= 57)** **Mean (SD)**	**Δ**	**Corrected** ***P*-value**
EORTC QLQ-C30 ^*a*^							
Global health status/ QoL	75.0 (15.5)	69.8 (19.0)	−5.2	75.3 (18.9)	75.7 (19.6)	0.4	.317
Functioning							
Physical functioning	82.2 (15.9)	84.8 (15.2)	2.6	86.8 (16.4)	87.3 (15.4)	0.5	.482
Role functioning	76.3 (23.9)	81.6 (23.7)	5.3	75.5 (28.4)	79.8 (26.1)	4.3	.968
Emotional functioning	81.3 (17.4)	79.3 (20.6)	−2	83.6 (18.2)	82.7 (19.8)	−0.9	.700
Cognitive functioning	87.9 (16.8)	89.1 (16.8)	1.2	85.6 (18.6)	83.9 (20.4)	−1.7	.417
Social functioning	82.8 (25.5)	87.4 (15.8)	4.6	83.1 (23.0)	86.0 (20.1)	2.9	.626
Symptoms							
Fatigue	33.3 (19.4)	28.7 (22.7)	−4.6	33.3 (28.4)	30.4 (26.4)	−2.9	.377
Pain	20.7 (25.4)	19.0 (23.9)	− 1.7	14.4 (24.4)	14.0 (24.2)	−0.4	.516
Nausea and vomiting	9.09 (17.2)	9.20 (15.2)	0.11	7.21 (13.0)	9.06 (14.8)	1.85	.537
Insomnia	28.3 (30.2)	31.0 (33.3)	2.7	19.4 (26.5)	23.4 (25.9)	4	.690
Dyspnoea	21.9 (23.4)	19.5 (24.4)	−2.4	14.4 (24.7)	14.0 (23.5)	−0.4	.589
Loss of appetite	16.2 (29.0)	16.1 (26.2)	−0.1	6.31 (18.9)	5.26 (15.2)	−1.05	.940
Constipation	16.7 (22.4)	9.52 (17.8)	−7.18	4.05 (13.5)	7.02 (18.6)	2.97	.064
Diarrhoea	20.2 (31.1)	18.5 (29.7)	−1.7	20.1 (25.3)	21.1 (28.6)	1	.332
Financial difficulties	8.08 (16.7)	5.75 (12.8)	−2.33	6.31 (20.4)	5.26 (15.2)	−1.05	.720
EORTC QLQ-CML24^*a*^							
**Impact on daily life**	**16.0 (15.2)**	**18.4 (19.1)**	**2.4**	**19.2 (17.1)**	**15.0 (15.8)**	**−4.2**	**.043**
**Symptom burden**	**25.6 (15.2)**	**22.5 (16.2)**	**−3.1**	**20.6 (12.4)**	**21.6 (13.2)**	**1**	**.013**
Impact on worry/mood	21.1 (20.8)	25.9 (22.5)	4.8	15.4 (13.9)	20.0 (18.5)	4.6	.671
Body image problems	28.1 (26.9)	20.7 (24.3)	−7.4	20.1 (27.2)	22.8 (29.7)	2.7	.052
Satisfaction with care and information	77.4 (24.9)	77.8 (17.3)	0.4	72.4 (32.0)	74.2 (30.9)	1.8	.871
Satisfaction with social life	67.7 (28.7)	64.4 (25.1)	−3.3	63.9 (33.2)	59.6 (33.2)	−4.3	.844
	**Questionnaire group**			**Intervention group**			
**Moderate to severe burden of following complaints (EORTC QLQ-CML24):**	**Pre (*n*** **= 33) %**	**Post (*n*** **= 29) %**	**Δ**	**Pre (*n*** **= 75) %**	**Post (*n*** **= 57) %**	**Δ**	**Corrected** ***P*-value**
Abdominal pain or cramps	9.4	3.4	−6	8.1	10.5	2.4	*
Dry mouth	21.9	27.6	5.7	12.2	19.3	7.1	.538
Worry about change in weight	12.5	6.9	−5.6	5.4	7.1	1.7	.365
Skin problems	31.3	24.1	−7.2	14.9	19.3	4.4	.170
Headache	3.1	6.9	3.8	4.1	7.0	2.9	.910
Aches or pains in muscles or joints	37.5	32.1	−5.4	25.7	33.9	8.2	.080
Hair loss	15.6	3.4	−12.2	5.4	3.5	−1.9	.254
Excessive sweating	19.4	10.3	−9.1	14.9	10.5	−4.4	.527
Acid indigestion or heartburn	9.4	13.8	4.4	8.1	8.9	0.8	.805
Drowsiness	15.6	3.4	−12.2	12.2	12.5	0.3	.072
Oedema	21.9	17.2	−4.7	16.2	12.3	−3.9	.946
Frequent urination	28.1	17.2	−10.9	29.7	19.3	−10.4	.639
Problem with eyes	28.1	20.7	−7.4	18.9	19.6	0.7	.479
Muscle cramps	37.5	34.5	−3	24.3	24.6	0.3	.433
Emotional ups and downs	12.5	13.8	1.3	9.6	14.3	4.7	.259
Worry about future health	28.1	20.7	−7.4	12.7	14.0	1.3	.137
Difficulty living a normal life because I got tired quickly	21.9	13.8	−8.1	17.8	15.8	−2	.529
Worry about infection	12.5	27.6	15.1	4.1	19.6	15.5	.373
Dissatisfied with my body as a result of illness or treatment	25.0	13.8	−11.2	15.5	17.5	2	.073
**Burdensome treatment**	**16.1**	**20.7**	**4.6**	**19.2**	**5.3**	**−13.9**	**.043**
Social support was needed	0.0	3.4	3.4	5.5	5.3	−0.2	*
Satisfied with the care	89.3	96.3	7	80.6	83.0	2.4	.278
Satisfied with the information	90.3	86.2	−4.1	76.8	83.6	6.8	.280
Satisfied about the quality of my social life	78.1	69.0	−9.1	68.5	63.2	−5.3	.668

*P*-value shows the difference between intervention and questionnaire with regard to the within-subject change between baseline and post-measurement

Δ = difference between pre- and post-measurements

*P-value could not be determined due to limited number in participants

^a^Scores between 0 and 100; higher scores on the functioning scales indicate better quality of life, higher scores on the symptom scales indicate more/more severe symptoms. Higher scores on daily life, symptom burden, worry/mood and body image problems, indicates higher impact, higher scores on satisfaction indicate more satisfaction

## Discussion

### Principal results and comparison with prior work

This is the first patient directed preference trial, including a before-after measurement, to explore the effectiveness of CMyLife on information provision, patient empowerment, medication compliance, molecular monitoring, and quality of life of CML patients. Overall, active use of CMyLife showed a clear trend towards improvement of all above-mentioned components except quality of life. The quality of life questionnaire showed a slight increase in patients’ burden of symptoms but simultaneously a decrease on impact on daily life.

Both familiarity with CML related concepts and perceived information provision showed positive trends in patients that actively used CMyLife and information on additional support outside the hospital improved significantly. Especially information provision on direct disease related concepts, such as diagnosis, treatment, tests, and side effects, showed a positive trend towards better information provision, although the percentage of sufficiently informed patients stayed unchanged low in other items. Content of CMyLife should be adjusted according to patients’ information needs to improve information provision. Previous literature emphasized the beneficial effects of adequate information provision in cancer patients [33–37]. Perceived information provision in patients with other malignancies varied although it is roughly comparable [38–40].

In addition to information provision, CMyLife significantly improved patients’ knowledge on where to find online health information. Literature shows that only half of cancer patients have knowledge and capability to access and differentiate the high amount of web-based information [41]. Results showed that active use of CMyLife improved eHealth literacy among CML patients and helped them become a well-informed partner in health care. Especially during the COVID-19 pandemic for instance, it is important for CML patients to stay accurately informed. Accurate and reliable information should be provided rapidly to these patients since almost 40% of them reported to be substantially concerned about the coronavirus [42]. CMyLife had been a great medium to provide patients with this timely information [43].

Patient empowerment was increased in our study, albeit not significant. Patients who are more empowered in taking control of their own disease could believe in their abilities to optimize their medication compliance and oversee their molecular monitoring. Other interactive patient portals, such as one for breast cancer survivors and one for lung cancer patients, showed a decrease in patient activation, although not directly comparable due to a difference in study population, content and features [44, 45]. Perhaps no significant increase in patient empowerment was found because of limited sample size, because the outcome measure is not responsive enough to detect an effect, or because actual usage was not strong enough. Therefore, future studies should take actual usage into account when investigating patient empowerment.

Our study showed extremely high medication compliance rates. Pre-intervention, these rates were reported as almost 100% and did not significantly change post-intervention. In literature, medication compliance rates vary from 19 to 98%, probably due to heterogeneity in adherence measurement methods and study populations [46, 47]. Only small proportions of patients were perfectly adherent. Patients in our study were very strict in taking their medication because they are very motivated for taking care of their disease, and therefore, participated in this patient preference study. As MARS is a rather general measure it is not possible to determine whether patients take their medication on all prescribed moments. Therefore, a more objective and specific tool to measure medication compliance should be used in the future. The well-known medication event monitoring system is an example of such an adequate measure, it provides an objective and reliable measure of medication compliance [48, 49]. Nevertheless, self-reported effectiveness shows patients to be more motivated and medication compliant when using the medication app.

Our study results show that frequency of hospital visits was not significantly affected by CMyLife. Remarkably, hospital visits decreased in both groups, probably due to the COVID-19 pandemic, during which routine care was deliberately postponed and digital care was stimulated. CMyLife enabled CML patients to continuate their care digitally without any delay or interruption. CMyLife gave patients opportunity to replace in-person visits by video consultations, which decreased the risk of exposing them to COVID-19 [50]. Self-reported effectiveness showed that by using the guideline app, patients felt more motivated to undergo testing on scheduled time, and they gained more insights in their obtained results and disease course. Importance of patient empowerment is further emphasized by suboptimal guideline adherence rates by HCPs [51]. CMyLife could be a great medium for accurate surveillance of treatment-free remission in CML patients.

None of the EORTC-QLQ-C30 scales showed significant differences in quality of life between the questionnaire and intervention group. Scores in our study are roughly comparable to cancer patients in general [52]. The EORTC-QLQ-CML24 scales showed a significant decrease of impact on daily life and a significant increase in symptom burden. Using CMyLife with logging their side effects could have made patients more aware of their symptoms. On the other side the concurrent decrease in impact suggests that patients were better able to self-manage their symptoms. In CML, adverse events are the main reason of intentional non-adherence, so timely recognition and management by HCPs is important [53–56]. Yet, HCPs tend to underestimate severity of patients’ symptoms [57]. Patient reported outcomes (PROs) are essential in patient-centered care, and its advantages as well as the necessity for routine PRO assessment in clinical practice are well described, not only in CML, but other malignancies too [57–61]. CMyLife provides a platform for this assessment.

### Future improvements

Despite promising potential of CMyLife, there is still room for improvement. In the future, content of CMyLife should be adjusted according to changing patients’ information needs. This requires a dynamic improvement process, where patients’ needs are measured routinely and content of CMyLife is adjusted accordingly. In addition to the information gap, PRO assessment combined with personalized feedback should be properly integrated in the CMyLife platform together with proper monitoring of molecular levels and medication adherence to enhance self-management aiming at the ultimate goal of true patient-centered care. HCPs will not become entirely redundant as monitoring and follow-up for adverse events and late effects of TKI treatment, albeit from a distance, is still required but their role is changing in coaching the patient.

### Strengths, limitations and future research

In our study a number of strengths and limitations are recognized. First, our results are strengthened by the presence of a questionnaire group. Despite the limited sample size and the sample being a convenience sample, it encompasses an adequate, nationwide, representation of Dutch CML patients. Our preference-based design has advantages compared to randomised controlled trials (RCTs). EHealth users should be motivated in order to actually use the innovation. Patients with a strong preference for one of the arms who refuse randomisation may be absent in RCTs. Also, literature shows high dropout and nonresponse rates in studies evaluating the effectiveness of eHealth using RCTs. When randomising patients to use or not use an eHealth innovation, bias will be introduced. Not all patients in the intervention group wanted to use the innovation and patients in the control group could have wanted to use the intervention but were not allowed to. Absence, dropout, and nonresponse of patients affects generalisability of study results, which is not the case in a preference based design [62–64]. Allocation of patients to use or not use eHealth randomly is therefore not ethical and should always be done in agreement with patients. However, when using a non-randomised design, unknown and uncontrolled confounders could be present [65]. Access to the CMyLife website for patients in the questionnaire group was not denied, this could have caused underestimation of the effects of the platform. Although as many covariates as possible were taken into account in our analysis, some factors should be focus of future studies in order to minimize confounding effects. For example, no data of CMyLife utilization were collected, nor how patients’ use other information and communication resources aside from CMyLife. Lastly, communication between patients could not have been prevented since patients in the intervention group met in small groups during workshops. Also, part of the platform was that patients in the intervention group were enabled to communicate with other patients in the intervention group, to share information or ask questions via a password protected forum. However, communication between the intervention group and the questionnaire group was very unlikely since the groups did not meet each other and had no contact information of each other. Therefore, we do not think this could have affected results.

## Conclusion

Aim of CMyLife is to provide hospital-free virtual care, by giving CML patients tools and know-how to self-manage their disease. Results of our study suggest that although more symptoms were reported, patient were better able to manage their symptoms. Knowledge on where to find online health information was improved significantly and, albeit not significant, they felt more empowered. Beneficial effects on medication compliance and molecular monitoring were experienced by patients, which is paramount in patient-centered care. CMyLife provides patients with information, tailored in amount and content and in a timely manner. However, we found room for improvement in content of information provision by expanding topics, adjusted to patients’ information needs. Personalized information based on PROs assessed in CMyLife such as symptoms experienced, and quality of life could further enhance self-management. An iterative process of assessing patients’ needs and further adjustment of CMyLife is required to keep care patient-centered, fit CMyLife into future perspectives, and put patients in lead of their disease process. Since hospital-free care has shown to be feasible in time of the COVID-19 pandemic, eHealth-based innovations such as CMyLife could be a solution to maintain quality of care and make current oncological health care services more sustainable.

## Supplementary Information

**Additional file 1.** Validated questionnaires with scores and internal consistency.

**Additional file 2.** Self-reported effectiveness on medication compliance and molecular monitoring questions and interpretation.

**Additional file 3.** Patients’ familiarity with CML related concepts questionnaire vs intervention group.

## Data Availability

The datasets used and/or analysed during the current study are available from the corresponding author on reasonable request.

## Abbreviations

CML: Chronic myeloid leukemia
eHEALS: eHealth literacy scale
HCP: Health care provider
MARS: Medication adherence rating scale
PAM: Patient activation measure
PRO: Patient reported outcome
RCT: Randomised controlled trail
TKI: Tyrosine kinase inhibitor
