# Supplementary material for: Effectiveness of digital care platform CMyLife for patients with chronic myeloid leukemia: results of a patient-preference trial
Source: BMC Health Serv Res. 2023 Mar 8;23:228. doi: 10.1186/s12913-023-09153-9 (PMC9994406; doi:10.1186/s12913-023-09153-9)
Supplement: Supplementary file 1 — Additional file 1. Validated questionnaires with scores and internal consistency. [file 12913_2023_9153_MOESM1_ESM.docx]

Additional file 1. validated questionnaires with scores and internal consistency.

The EORTC QLQ-INFO25 (scores between 0-100 with higher scores indicating more/better information and satisfaction) (internal consistency of α = 0.74-0.89).

The eHealth Literacy Scale (eHEALS) (scores between 0-5 with higher scores indicating better eHealth literacy) (internal consistency of α = 0.93).

The Patient Activation Measure (PAM) (level 1-4 and scores between 0-100 with higher levels and scores indicating better patient activation) (internal consistency of α = 0.88).

The Medication Adherence Rating Scale (MARS) (scores between 5-25 with lower scores indicating better medication compliance) (internal consistency of α = 0.76).

The EORTC QLQ-C30 (scores between 0-100, higher scores on the functioning scales mean better quality of life, higher scores on the symptom scales mean more/more severe symptoms) (internal consistency of *α* = 0.65-0.92).

The EORTC QLQ-CML24 (scores between 0-100, higher scores on daily life, symptom burden, worry/mood and body image problems, means higher impact, higher scores on satisfaction mean more satisfaction) (internal consistency of α = 0.66-0.83).
