# Supplementary material for: Effectiveness of digital care platform CMyLife for patients with chronic myeloid leukemia: results of a patient-preference trial
Source: BMC Health Serv Res. 2023 Mar 8;23:228. doi: 10.1186/s12913-023-09153-9 (PMC9994406; doi:10.1186/s12913-023-09153-9)
Supplement: Supplementary file 2 — Additional file 2. Self-reported effectiveness on medication compliance and molecular monitoring questions and interpretation. [file 12913_2023_9153_MOESM2_ESM.docx]

Additional file 2. Self-reported effectiveness on medication compliance and molecular monitoring questions and interpretation.

| The influence of the medication app on medication adherence | Completely disagree | Disagree | Neutral | Agree | Completely agree |
| --- | --- | --- | --- | --- | --- |
| When using the medication app I know better why it is important to take medication properly |  |  |  |  |  |
| The medication app makes me more aware of the importance to take medication properly |  |  |  |  |  |
| The medication app motivates me to take medication properly |  |  |  |  |  |
| The medication app encourages to seek help (if necessary) to take medication properly |  |  |  |  |  |
| The medication app has improved my medication adherence |  |  |  |  |  |
| The medication app makes me feel less insecure about my medication adherence |  |  |  |  |  |

| The influence of the guideline app on guideline adherence | Completely disagree | Disagree | Neutral | Agree | Completely agree |
| --- | --- | --- | --- | --- | --- |
| I have more insight into my BCR-ABL1 value and its course |  |  |  |  |  |
| I know better whether my treatment is working |  |  |  |  |  |
| I know better when to have my BCR-ABL1 value checked |  |  |  |  |  |
| The guideline app makes more aware of importance of checking BCR-ABL1 value in time |  |  |  |  |  |
| The guideline app motivates to have BCR-ABL1 value checked in time |  |  |  |  |  |
| The guideline app clearly shows me what my BCR-ABL1 values mean |  |  |  |  |  |
| The guideline app encourages to contact HCPs if treatment response is not sufficient |  |  |  |  |  |
| The guideline app makes me feel less insecure about my BCR-ABL1 values |  |  |  |  |  |

For the interpretation of the results completely disagree and disagree were grouped and completely agree and agree were grouped.
