# Supplementary material for: Effectiveness of digital care platform CMyLife for patients with chronic myeloid leukemia: results of a patient-preference trial
Source: BMC Health Serv Res. 2023 Mar 8;23:228. doi: 10.1186/s12913-023-09153-9 (PMC9994406; doi:10.1186/s12913-023-09153-9)
Supplement: Supplementary file 3 — Additional file 3. Patients’ familiarity with CML related concepts questionnaire vs intervention group. [file 12913_2023_9153_MOESM3_ESM.docx]

Additional file 3. Patients' familiarity with CML related concepts questionnaire vs intervention group.

|  | Questionnaire group | | | Intervention group | | |  |
| --- | --- | --- | --- | --- | --- | --- | --- |
| Not (at all) familiar with undermentioned concepts: | **Pre (n=33) %** | **Post (n=29) %** | **Δ** | **Pre (n=75)**  **%** | **Post (n=57)**  **%** | **Δ** | **Corrected *P*-value** |
| Philadelphia chromosome (t9;22) | 25.1 | 20.7 | -4.4 | 12.3 | 8.9 | -3.4 | * |
| BCR-ABL1 | 20.0 | 7.4 | -12.6 | 12.3 | 5.3 | -7 | .323 |
| Tyrosine Kinase Inhibitor | 33.3 | 29.6 | -3.7 | 26.0 | 8.9 | -17.1 | .054 |
| Remission | 40.0 | 40.7 | 0.7 | 30.6 | 18.5 | -12.1 | .177 |
| Log reduction (for BCR-ABL1) | 62.1 | 63.0 | 0.9 | 57.5 | 49.1 | -8.4 | .440 |
| Hematological response/remission | 46.7 | 48.1 | 1.4 | 44.4 | 32.1 | -12.3 | .218 |
| Cytogenetic response/remission | 69.0 | 66.5 | -2.5 | 59.2 | 50.9 | -8.3 | .360 |
| Molecular response/remission | 67.9 | 59.3 | -8.6 | 47.2 | 34.5 | -12.7 | .879 |
| Major Molecular Remission (MMR) | 67.9 | 61.6 | -6.3 | 60.6 | 50.0 | -10.6 | .794 |
| Complete Cytogenetic Remission (CCyR) | 72.4 | 57.7 | -14.7 | 63.4 | 55.6 | -7.8 | .265 |
| Treatment-free Remission (TFR) | 82.1 | 65.4 | -16.7 | 68.1 | 49.1 | -19 | .639 |
| Hematon | **44.8** | **34.6** | **-10.2** | **42.3** | **18.2** | **-24.1** | **.023** |

*P*-value shows the difference between intervention and questionnaire with regard to the within-subject change between baseline and post-measurement.

Δ= difference between pre- and post-measurements.

**P*-value could not be determined due to limited number of participants.
